# The effect of inhaler prescription on the development of lung cancer in COPD: a nationwide population-based study

**DOI:** 10.1186/s12931-024-02838-7

**Published:** 2024-05-31

**Authors:** Ji Eun Park, Eunyoung Lee, Dave Singh, Eun Kyung Kim, Bumhee Park, Joo Hun Park

**Affiliations:** 1 Department of Pulmonary and Critical Care Medicine, Ajou University School of Medicine, Worldcup-ro 164, Suwon, Gyeonggi-do 16499 Republic of Korea; 2 Department of Neurology, McGovern Medical School at UTHealth, Houston, TX US; 3 Division of Infection, Immunity and Respiratory Medicine, The University of Manchester and Manchester University NHS Foundation Trust, Manchester, UK; 4 Department of Pulmonology, Allergy and Critical Care Medicine, CHA Bundang Medical Center, CHA University, Seongnam, Republic of Korea; 5 Office of Biostatistics, Medical Research Collaborating Center, Ajou Research Institute for Innovative Medicine, Ajou University Medical Center, Suwon, Republic of Korea; 6 Department of Biomedical Informatics, Ajou University School of Medicine, Suwon, Republic of Korea

## Abstract

**Background:**

COPD is associated with the development of lung cancer. A protective effect of inhaled corticosteroids (ICS) on lung cancer is still controversial. Hence, this study investigated the development of lung cancer according to inhaler prescription and comorbidties in COPD.

**Methods:**

A retrospective cohort study was conducted based on the Korean Health Insurance Review and Assessment Service database. The development of lung cancer was investigated from the index date to December 31, 2020. This cohort included COPD patients (≥ 40 years) with new prescription of inhalers. Patients with a previous history of any cancer during screening period or a switch of inhaler after the index date were excluded.

**Results:**

Of the 63,442 eligible patients, 39,588 patients (62.4%) were in the long-acting muscarinic antagonist (LAMA) and long-acting β2-agonist (LABA) group, 22,718 (35.8%) in the ICS/LABA group, and 1,136 (1.8%) in the LABA group. Multivariate analysis showed no significant difference in the development of lung cancer according to inhaler prescription. Multivariate analysis, adjusted for age, sex, and significant factors in the univariate analysis, demonstrated that diffuse interstitial lung disease (DILD) (HR = 2.68; 95%CI = 1.86–3.85), a higher Charlson Comorbidity Index score (HR = 1.05; 95%CI = 1.01–1.08), and two or more hospitalizations during screening period (HR = 1.19; 95%CI = 1.01–1.39), along with older age and male sex, were independently associated with the development of lung cancer.

**Conclusion:**

Our data suggest that the development of lung cancer is not independently associated with inhaler prescription, but with coexisting DILD, a higher Charlson Comorbidity Index score, and frequent hospitalization.

**Supplementary Information:**

The online version contains supplementary material available at 10.1186/s12931-024-02838-7.

## Introduction

Several epidemiologic studies suggest a close association between chronic obstructive pulmonary disease (COPD) and lung cancer [1–4]. COPD even in never smokers is associated with lung cancer, and the presence of COPD in smokers is associated with a two to six times higher risk for the development of lung cancer [1, 5, 6].

Pathogenic mechanisms for the association between COPD and lung cancer comprise cigarette smoking, the increased expression of growth factors in COPD, chronic inflammation, genetic predisposition, epigenetic mechanism, and premature aging [7, 8]. Furthermore, some comorbidities including diabetes mellitus and tuberculosis in COPD are reported to be risk factors of lung cancer [6, 9].

Recently, pharmacological treatment with inhaled corticosteroids (ICS) was suggested as a strategy to reduce the risk of lung cancer, since chronic inflammation in COPD promotes tumor growth and suppresses antitumor immune responses [10, 11]. Retrospective meta-analyses have shown that ICS lowers the risk of lung cancer in COPD, although the quality of the evidence is low [12, 13]. However, some studies failed to confirm the link between ICS and lung cancer [13, 14]. Time-related biases, including immortal time bias, latency time bias, and protopathic bias, were not fully accounted for in previous studies, leading to conflicting results. Moreover, the effects of other anti-inflammatory therapies including long-acting muscarinic antagonist (LAMA) and long-acting β2-agonist (LABA) therapy on the development of lung cancer in COPD remain to be determined.

We investigated the development of lung cancer in COPD according to inhaler prescription and comorbidities by analyzing the Korean Health Insurance Review and Assessment Service (HIRA) database. The study design used accounted for time-related biases, to provide further information regarding the risks of lung cancer development using a large sample size.

## Materials and methods

### Study design

This study analyzed the data from the HIRA database from January 1, 2015, to December 31, 2020. The HIRA database contains medical service claims records including all diagnoses and medications from all medical care settings for almost the entire Korean population under mandatory and universal national health insurance.

**Fig. 1:**
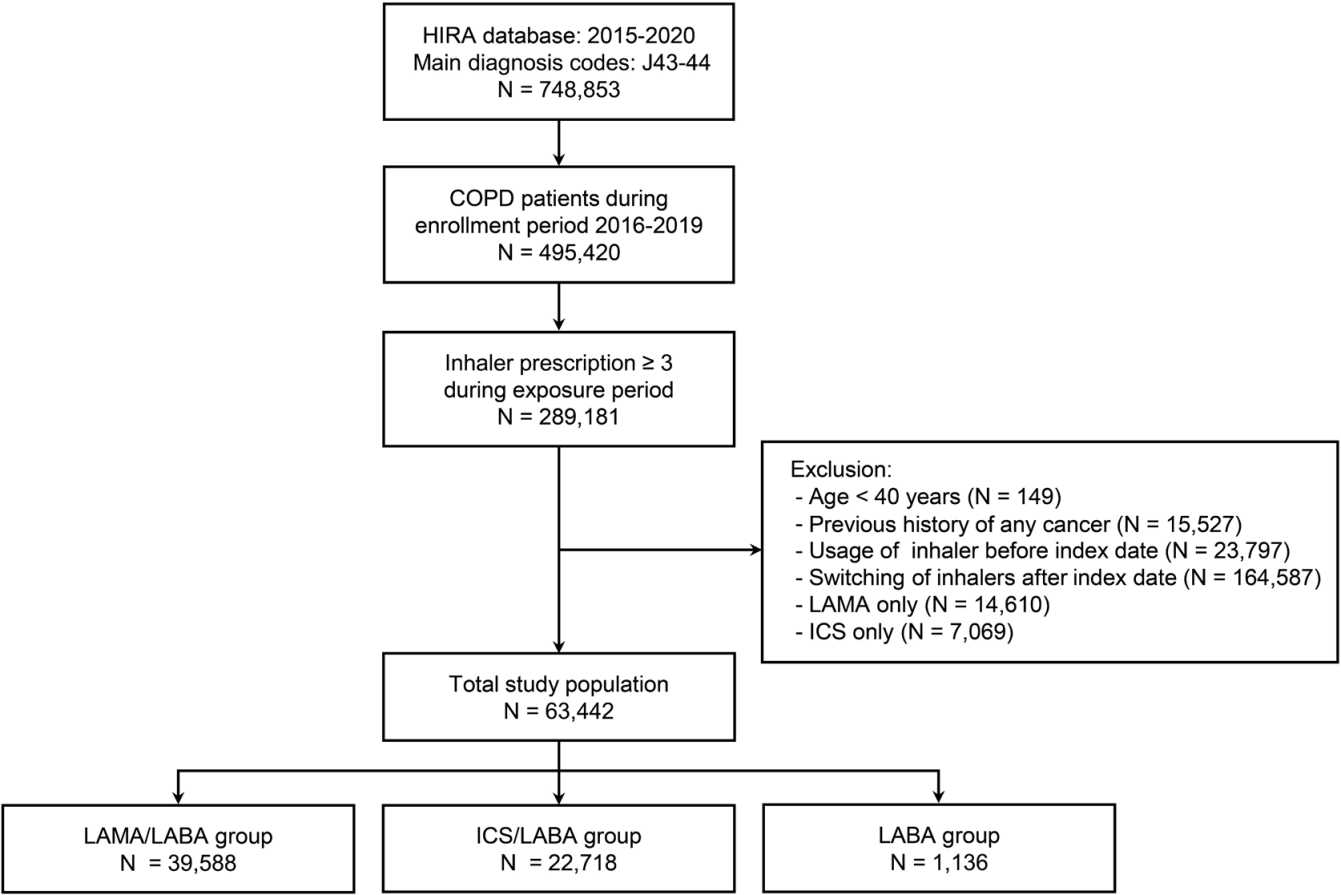
Flow chart of study population. Abbreviations: CCI = Charlson Comorbidity Index score; COPD = chronic obstructive pulmonary disease; HIRA = Health Insurance Review and Assessment Service; ICS = inhaled corticosteroid; LABA = long-acting beta-agonist; LAMA = long-acting muscarinic antagonist

The COPD cohort out of the HIRA database was constructed by the following criteria: (1) patients aged ≥ 40 years, (2) at least three separate outpatient visits, (3) COPD (J43⎼J44 of International Classification of Diseases tenth revision (ICD-10) codes) as the primary diagnosis from January 1, 2015, to December 31, 2020, with the prescription of one of the following respiratory medications ; LAMA, LABA, combination of LAMA/LABA, ICS, combination of ICS/LABA, triple therapy (LAMA + LABA + ICS), phosphodiesterase-4 inhibitors, theophylline, and mucolytics (Fig. 1). Three or more prescriptions of an inhaler during the exposure period were required for being enlisted into each inhaler group. The oral corticosteroid (OCS) usage was identified as the prescription of prednisolone 420 mg (15 mg/day for four weeks) or more for COPD during the exposure period.

**Fig. 2:**
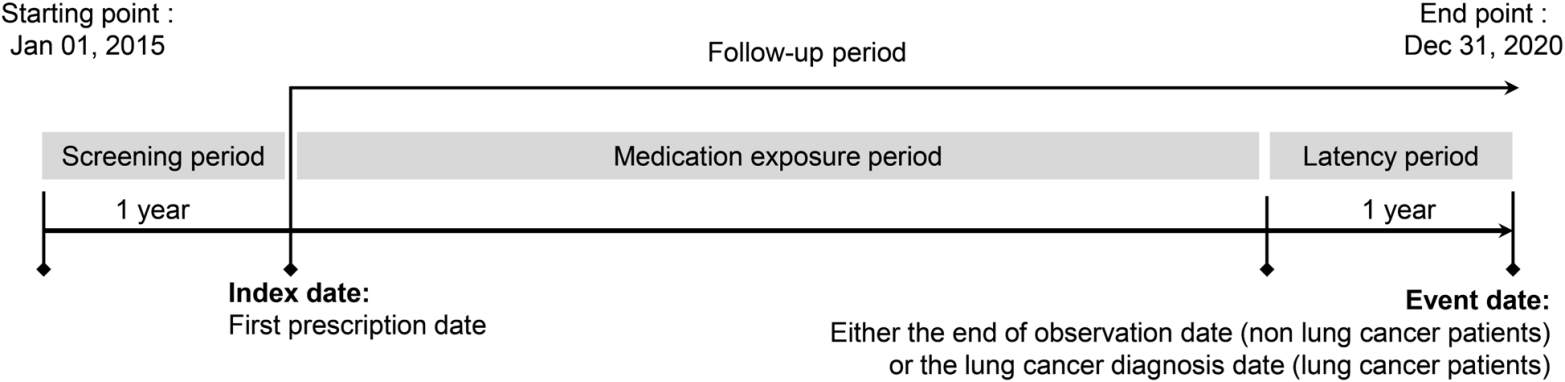
Study design. Medications received during the latency period between medication exposure and lung cancer diagnosis were not counted as exposures

Each patient had one-year screening period without any inhaler medication before the index date. The index date was defined as the date of the first prescription for inhaler medication. A latency period before lung cancer diagnosis was set to allow sufficient time for inhaler exposure with regard to cancer development, as in other studies [14, 15] (Fig. 2).

During the screening period, subjects who had any cancer history or who had been prescribed an inhaler were excluded. Patients who had prescription switched between inhaler medications after the index date were also excluded.

This cohort consisted of three groups: 1) LAMA/LABA group as LAMA + LABA or LAMA/LABA fixed-dose combination, 2) ICS/LABA group as ICS + LABA or ICS/LABA fixed-dose combination, 3) LABA group using a LABA inhaler alone. The subjects were monitored for the diagnosis of lung cancer from January 1, 2016 to December 31, 2020 (Fig. 2).

### Case identification

Cases of lung cancer (C33–C34) were identified by ICD-10 codes after the initial prescription of inhalers. Comorbidities were also identified based on following ICD-10 codes: asthma (J45-46), hypertension (I10-15), diabetes mellitus (E10–E14), diffuse interstitial lung disease (DILD) (J84), ischemic heart disease (I20–I25), heart failure (I50), cerebrovascular disease (I60–I69), and pulmonary thromboembolism (I26). The event date was the first date of cancer diagnosis based on the above ICD-10 codes. Patients in whom lung cancer was diagnosed in the latency period after the initial prescription were excluded.

### Adjustment for covariates

Multivariate model analyses were performed including covariates affecting the risk of cancer development. Adjustment for the severity of COPD using the Charlson Comorbidity Index and the number of emergency room visits and hospitalizations was performed. The multivariate analyses included two models: Model 1 had all covariates and model 2 had covariates including age, sex, and significant factors in the univariate analysis.

### Statistical analysis

Baseline characteristics and the prescription of medications were summarized by descriptive statistics including mean, standard deviation, and proportion. A chi-squared test was used for categorical variables, and a one-way analysis of variance (ANOVA) was used for continuous variables. The prevalence of lung cancer among the three groups according to inhaler therapy was tested by a chi-squared test and adjusted by a Bonferroni correction for multiple comparisons. Incidence rate of lung cancer per 10,000 person-years were computed with 95% confidence intervals (CIs) and compared with the Poisson regression analysis. The proportional hazard assumption was analyzed using Schoenfeld residuals for the Cox proportional hazards regression model. Univariate and multivariate Cox proportional hazards regression analyses were used to identify significant risk factors predicting the development of lung cancer.

Sensitivity analyses conducted by setting latency periods of 6 months, 12 months, and 24 months were performed to determine the effect of protopathic bias. When calculating the cancer risk, the inhaler medication used during the latency period was not considered. Hazard ratio (HR) with 95% CI was assessed for the risk of lung cancer. The analysis was performed only on cases with complete data. A threshold of *p* < 0.05 was deemed significant. All analyses were performed using SAS version 9.4 (SAS Institute, Cary, NC, USA).

### Ethics statement

The present study was approved by the Institutional Review Board of Ajou University Hospital (AJOUIRB-EXP-2021-582). The requirement for informed consent from the patients studied was waived by the ethical review board.

## Results

### Baseline characteristics

This cohort comprised 63,442 patients with a mean age of 69.1 years (75.7% male; Table 1). A total of 39,588 patients (62.4%) were categorized in the LAMA/LABA group, 22,718 (35.8%) in the ICS/LABA group, and 1,136 (1.8%) in the LABA group (Table 1). The mean age of the ICS/LABA group was younger, and the proportion of women in the ICS/LABA group was higher (*p* < 0.001) (Table 1). Among the comorbidities during the screening period, asthma was significantly more co-existent in the ICS/LABA group. Diabetes, DILD, ischemic heart disease, heart failure, and cerebrovascular diseases were more frequently observed in the LAMA/LABA group (*p* < 0.001) (Table 1). Accordingly, the Charlson Comorbidity Index score was higher in the LAMA/LABA group (*p <* 0.001) (Table 1).

**Table 1:** Baseline characteristics of the cohort

	Total	LAMA/LABA	ICS/LABA	LABA	*P*-value
**Number**	63,442	39,588	22,718	1,136	
**Age (years)**	69.08 ± 10.20	69.33 ± 9.560)	68.62 ± 11.23	69.84 ± 9.68	< 0.001
**Age distribution (years), N (%)**					< 0.001
40–49	2,326 (3.67)	996 (2.52)	1,299 (5.72)	31 (2.73)	
50–59	9,195 (14.49)	5,354 (13.52)	3,702 (16.3)	139 (12.24)	
60–69	19,731 (31.10)	12,893 (32.57)	6,467 (28.47)	371 (32.66)	
70–79	22,088 (34.82)	14,533 (36.71)	7,150 (31.47)	405 (35.65)	
80–	10,102 (15.92)	5,812 (14.68)	4,100 (18.05)	190 (16.73)	
**Male sex, N (%)**	47,994 (75.65)	32,530 (82.17)	14,540 (64)	924 (81.34)	< 0.001
**Co-morbidities during screening period, N (%)**					
Asthma (J45–J46)	30,630 (48.28)	15,598 (39.4)	14,664 (64.55)	368 (32.39)	< 0.001
Hypertension (I10–I15)	33,857 (53.37)	21,247 (53.67)	12,025 (52.93)	585(51.50)	0.091
Diabetes mellitus (E10–E14)	15,740 (24.81)	10,119 (25.56)	5,358 (23.58)	263 (23.15)	< 0.001
Diffuse interstitial lung disease (J84)	501 (0.79)	355 (0.90)	138 (0.61)	8 (0.70)	< 0.001
Ischemic heart disease (I20–I25)	11,333 (17.86)	7,477 (18.89)	3,677 (16.19)	179 (15.76)	< 0.001
Cerebrovascular disease (I60–I69)	7,964 (12.55)	5,145 (13.00)	2,680 (11.80)	139 (12.24)	< 0.001
Heart failure (I50)	5,219 (8.23)	3,429 (8.66)	1,729 (7.61)	61 (5.37)	< 0.001
Pulmonary embolism (I26)	253 (0.40)	163 (0.41)	87 (0.38)	3 (0.26)	0.661
**Charlson Comorbidity Index score**	2.73 ± 1.92	2.74 ± 1.95	2.71 ± 1.88	2.53 ± 1.83	< 0.001
**Charlson Comorbidity Index score, N (%)**					< 0.001
0 (CCI score = 0)	3,529 (5.56)	2,441 (6.17)	987 (4.34)	101 (8.89)	
1 (CCI score = 1)	15,821 (24.94)	9,643 (24.36)	5,901 (25.97)	277 (24.80)	
2 (CCI score = 2)	15,136 (23.86)	9,213 (23.27)	5,637 (24.81)	286 (25.18)	
3 (CCI score ≥ 3)	28,956 (45.64)	18,291 (46.2)	10,193 (44.87)	472 (41.55)	
**Hospital visit during screening period, N (%)**					
Hospitalization					
Respiratory related (J00–J99)					< 0.001
0	53,729 (84.69)	33,719 (85.17)	19,009 (83.67)	1,001 (88.12)	
1	6,867 (10.82)	4,241 (10.71)	2,523 (11.11)	103 (9.07)	
≥ 2	2,846 (4.49)	1,628 (4.11)	1,186 (5.22)	32 (2.82)	
Cardiovascular disease related (I00–I99)					< 0.001
0	58,695 (92.52)	36,256 (91.58)	21,366 (94.05)	1,073 (94.45)	
1	3466 (5.46)	2419 (6.11)	998 (4.39)	49 (4.31)	
≥ 2	1281 (2.02)	913 (2.31)	354 (1.56)	14 (1.23)	
Any reason					< 0.001
0	38,311 (60.39)	23,496 (59.35)	14,079 (61.97)	736 (64.79)	
1	13,604 (21.44)	8,831 (22.31)	4,534 (19.96)	239 (21.04)	
≥ 2	11,527 (18.17)	7,261 (18.34)	4,105 (18.07)	161 (14.17)	
Emergency room visit					
Respiratory related (J00–J99)					0.004
0	57,842 (91.17)	35,997 (90.93)	20,790 (91.51)	1,055 (92.87)	
1	4554 (7.18)	2950 (7.45)	1537 (6.77)	67 (5.90)	
≥2	1046 (1.65)	641 (1.62)	391 (1.72)	14 (1.23)	
Cardiovascular disease related (I00–I99)					< 0.001
0	61,308 (96.64)	38,063 (96.15)	22,133 (97.42)	1,112 (97.89)	
1	1776 (2.80)	1283 (3.24)	473 (2.08)	20 (1.76)	
≥ 2	358 (0.56)	242 (0.61)	112 (0.49)	4 (0.35)	
Any reason					< 0.001
0	48,281 (76.10)	29,673 (74.95)	17,699 (77.91)	909 (80.02)	
1	10,456 (16.48)	6876 (17.37)	3416 (15.04)	164 (14.44)	
≥ 2	4705 (7.42)	3039 (7.68)	1603 (7.06)	63 (5.55)	

The percentage is provided in parentheses

*Abbreviations: CCI *Charlson Comorbidity Index score, *ICS *Inhaled corticosteroids, *LABA *long-acting beta2-agonist, *LAMA *Long-acting muscarinic antagonist

The LAMA/LABA group and ICS/LABA group had more frequent hospitalizations than the LABA group, along with a higher rate of hospitalization for respiratory disease in the ICS/LABA group, and a higher rate of hospitalization for cardiovascular disease in the LAMA/LABA group (*p* < 0.001) (Table 1).

### Medications

During the exposure period, xanthine and mucolytics (54.8% and 75.0%, respectively) were dominantly prescribed in this cohort, whereas only 1.59% of the patients were prescribed roflumilast (Table 2). The OCS prescription for COPD was highest in the ICS/LABA group among the three groups (*p* < 0.001) (Table 2).

**Table 2:** Medication in this cohort

	Total	LAMA/LABA	ICS/LABA	LABA	*P*-value
**Number**	63,442	39,588	22,718	1,136	
**Inhaler**					
**Medication exposure period (median years) [IQR]**	1.84 [0.92–2.84]	1.71 [0.84–2.68]	2.08 [1.07–3.06]	2.66 [1.49–3.47]	< 0.001
LAMA + LABA		15,430 (38.98)			
LAMA/LABA (fixed dose)		24,158 (61.02)			
ICS + LABA			16,458 (72.44)		
ICS/LABA (fixed dose)			6,260 (27.56)		
LABA				1,136 (100.00)	
**Oral medication**					
Roflumilast	1,007 (1.59)	785 (1.98)	213 (0.94)	9 (0.79)	< 0.001
Xanthine	34,747 (54.77)	20,357 (51.42)	13,935 (61.34)	455 (40.05)	< 0.001
Mucolytics	47,558 (74.96)	28,249 (71.36)	18,597 (81.86)	712 (62.68)	< 0.001
Oral corticosteroid (≥ prednisolone 420 mg)	11,019 (17.37)	5,431 (13.72)	5,476 (24.10)	112 (9.86)	< 0.001

The percentage is provided in parentheses

*Abbreviations: IQR *interquartile range, *ICS *inhaled corticosteroids, *LABA *long-acting beta2-agonist, *LAMA *Long-acting muscarinic antagonist

### Prevalence and incidence of lung cancer

Among the three groups, the ICS/LABA group had the lowest five-year prevalence of lung cancer (*p* = 0.031) (Table 3). The incidence rate of lung cancer per 10,000 person-years was lower in the ICS/LABA group compared to the LAMA/LABA group and the LABA group (*p* < 0.001) (Table 3).

**Table 3:** Incidence and prevalence of lung cancer

	LAMA/LABA	ICS/LABA	LABA	Comparison among three groups	Comparison between LAMA/LABA and ICS/LABA	Comparison between LAMA/LABA and LABA	Comparison between ICS/LABA and LABA
				*P*-value	*P*-value*	*P*-value*	*P*-value*
**Incidence of lung cancer (C33–C34) per 10,000 person year [95% CI]**	85.5 [85.46–85.57]	72.56 [72.49–72.62]	99.4 [99.05–99.68]	< 0.001	< 0.001	< 0.001	< 0.001
Follow-up duration (median years) [IQR]	2.73 [1.86–3.70]	3.1 [2.10–4.07]	3.69 [2.54–4.49]	< 0.001	< 0.001	< 0.001	< 0.001
**Prevalence of lung cancer (C33–C34) for 5 years, N (%)**	934 (2.36)	500 (2.20)	38 (3.35)	0.031	0.613	0.096	0.034

The percentage is provided in parentheses

*Abbreviations*: *CCI* Charlson Comorbidity Index score, *CI* confidential interval, *ICS* inhaled corticosteroids, *IQR* interquartile range, *LABA* long-acting beta2-agonist, *LAMA* Long-acting muscarinic antagonist, *N* the number of patients

### Risk factors for the development of lung cancer

In multivariate model 1 adjusting for all the covariates, the risk of lung cancer was not statistically different in the LAMA/LABA group (Hazard ratio HR = 0.92; 95% CI = 0.67–1.28) and the ICS/LABA group (HR = 0.90; 95% CI = 0.65–1.26) compared to the LABA group (Table 4). Similar results were obtained in sensitivity analyses with 6-month, 12-month, and 24-month latency periods (Table 5). Furthermore, an effect modification analysis was undertaken to ascertain potential disparities in the effects of significant risk factors identified through multivariate analysis. Nevertheless, no statistically significant interaction was observed (Supplement Table 1). In multivariate model 2 adjusted for age, sex, and significant factors in the univariate analysis, independent associations with the development of lung cancer were observed for DILD (HR, 2.68; 95% CI, 1.86–3.85), a higher Charlson Comorbidity Index score (HR, 1.05; 95% CI, 1.01–1.08), and two or more hospitalizations during screening period (HR, 1.19; 95% CI, 1.01–1.39), along with male sex and older age (Table 4).

**Table 4:** Risk factors for the development of lung cancer in COPD

	Cox regression analysis					
	Univariate		Multivariate Model 1		Multivariate Model 2	
	Hazard ratio (95% CI)	*P*-value	Hazard ratio (95% CI)	*P*-value	Hazard ratio (95% CI)	*P*-value
Group						
LAMA/LABA	0.93 (0.67–1.28)	0.645	0.92 (0.67–1.28)	0.628	0.92 (0.67–1.28)	0.627
ICS/LABA	0.76 (0.54–1.05)	0.098	0.90 (0.65–1.26)	0.552	0.93 (0.67–1.29)	0.665
LABA	Ref.		Ref.		Ref.	
Age group						
40–49	Ref.		Ref.		Ref.	
50–59	2.04 (1.06–3.94)	0.034	1.90 (0.99–3.68)	0.056	1.90 (0.99–3.68)	0.055
60–69	5.19 (2.77–9.72)	< 0.001	4.66 (2.48–8.73)	< 0.001	4.66 (2.48–8.73)	< 0.001
70–79	7.87 (4.22–14.69)	< 0.001	7.17 (3.83–13.43)	< 0.001	7.15 (3.82–13.39)	< 0.001
80-	8.39 (4.47–15.75)	< 0.001	8.22 (4.37–15.48)	< 0.001	8.14 (4.32–15.33)	< 0.001
Male sex	2.65 (2.26–3.11)	< 0.001	2.75 (2.34–3.24)	< 0.001	2.73 (2.32–3.21)	< 0.001
Asthma	1.09 (0.98–1.21)	0.100	1.11 (1.00–1.24)	0.049		
Hypertension	1.16 (1.04–1.28)	0.005	0.92 (0.82–1.03)	0.126	0.90 (0.80–1.01)	0.068
Diabetes mellitus	1.21 (1.08–1.35)	0.001	0.98 (0.85–1.12)	0.738	1.01 (0.88–1.16)	0.935
Diffuse interstitial lung disease	3.14 (2.19–4.50)	< 0.001	2.64 (1.84–3.79)	< 0.001	2.68 (1.86–3.85)	< 0.001
Ischemic heart disease	1.15 (1.01–1.31)	0.030	0.95 (0.83–1.10)	0.512	0.92 (0.80–1.06)	0.236
Total stroke	1.13 (0.97–1.31)	0.118	0.85 (0.72–1.00)	0.047		
Heart failure	0.95 (0.78–1.16)	0.604	0.75 (0.61–0.93)	0.009		
Pulmonary embolism	0.55 (0.18–1.71)	0.301	0.48 (0.15–1.49)	0.203		
Charlson Comorbidity Index score	1.08 (1.06–1.11)	< 0.001	1.06 (1.02–1.10)	0.001	1.05 (1.01–1.08)	0.012
Hospitalization during screening period for any reason						
0	Ref.		Ref.		Ref.	
1	1.22 (1.08–1.38)	0.002	1.12 (0.98–1.28)	0.110	1.12 (0.98–1.29)	0.099
≥2	1.33 (1.17–1.52)	< 0.001	1.19 (1.02–1.40)	0.030	1.19 (1.01–1.39)	0.034
Emergency room visit during screening period for any reason						
0	Ref.		Ref.		Ref.	
1	1.17 (1.02–1.34)	0.024	1.02 (0.88–1.18)	0.798	1.02 (0.88–1.18)	0.837
≥2	1.24 (1.03–1.50)	0.027	1.00 (0.81–1.24)	0.986	0.99 (0.80–1.22)	0.917

Multi-variate cox regression analyses were performed in two models: Model 1 (including all factors), Model 2 (adjusted for age, sex, and significant factors in the univariate analysis)

*Abbreviations: CCI *Charlson Comorbidity Index score, *CI *Confidential interval, *COPD *Chronic obstructive pulmonary disease, *ICS *Inhaled corticosteroids, *LABA *Long-acting beta2-agonist, *LAMA *Long-acting muscarinic antagonist, *Ref *Reference

**Table 5:** Sensitivity analyses of the latency period

Latency period	Group	*N*	Lung cancer *N* (%)	Multivariate model 1		Multivariate model 2	
				Hazard ratio (95% CI)	*P*-value	Hazard ratio (95% CI)	*P*-value
0 month	LAMA/LABA	41,450	1,594 (3.85)	1.00 (0.75–1.32)	0.971	1.00 (0.75–1.32)	0.979
	ICS/LABA	23,725	798 (3.36)	0.97(0.73–1.29)	0.836	1.00 (0.75–1.33)	0.999
	LABA	1,084	50 (4.61)	Ref.		Ref.	
6 months	LAMA/LABA	40,668	1,172 (2.88)	0.85 (0.63–1.14)	0.269	0.85 (0.63–1.14)	0.270
	ICS/LABA	23,298	592 (2.54)	0.81 (0.60–1.09)	0.170	0.84 (0.62–1.13)	0.250
	LABA	1,101	47 (4.27)	Ref.		Ref.	
1 year	LAMA/LABA	39,588	934 (2.36)	0.92 (0.67–1.28)	0.628	0.92 (0.67–1.28)	0.627
	ICS/LABA	22,718	500 (2.20)	0.90 (0.65–1.26)	0.552	0.93 (0.67–1.29)	0.665
	LABA	1,136	38 (3.35)	Ref.		Ref.	
2 years	LAMA/LABA	28,383	476 (1.68)	0.96 (0.64–1.42)	0.826	0.95 (0.64–1.41)	0.800
	ICS/LABA	18,741	292 (1.56)	0.94 (0.63–1.41)	0.765	0.95 (0.64–1.42)	0.798
	LABA	1,114	26 (2.33)	Ref.		Ref.	

Multi-variate cox regression analyses were performed in two models: Model 1 (including all factors), Model 2 (adjusted for age, sex, and significant factors in the univariate analysis)

*Abbreviations: CI* Confidential interval, *ICS *Inhaled corticosteroids, *LABA *Long-acting beta2-agonist, *LAMA *Long-acting muscarinic antagonist, *Ref *Reference

## Discussion

Our study was performed to identify the risk of lung cancer associated with inhaler prescription and comorbidities in COPD. The study was designed to minimize time-related biases. Although the incidence of lung cancer was lower in the ICS/LABA group, multivariate analyses showed that the development of lung cancer was not associated with inhaler therapy but with DILD, a higher Charlson Comorbidity Index score, and two or more hospitalizations during screening period.

Our study used the HIRA database to analyze the development of lung cancer in COPD using a design based on the new initiation of LAMA/LABA, ICS/LABA, and LABA. Our multivariate analyses showed no significant difference in the development of lung cancer according to inhaler prescription. Several studies in COPD patients reported an association between ICS and a lower incidence of lung cancer [15–19], possibly attributed to a preventative role against lung cancer through anti-inflammatory effects [20]. Furthermore, Parimon et al. reported a dose-dependent reduced risk of lung cancer associated with ICS [16]. A recent study using a population-based cohort of COPD suggested that ICS usage was associated with a 30% decrease in the risk of lung cancer and a 43% reduction of lung cancer per gram of ICS use [15].

In contrast, some studies found no association between ICS therapy and lung cancer risk, compatible with our results [21–23]. A recent large cohort study reported no reduction of lung cancer incidence associated with ICS use in COPD patients [14]. There was no relationship between the duration and dosage of ICS therapy and the risk of lung cancer [14]. The authors pointed out that time-related biases, including immortal time bias, latency time bias and protopathic bias, and the inclusion of asthmatics may have influenced the studies previously reporting that ICS was associated with a reduced incidence of lung cancer [14].

The current analysis attempted to overcome the methodological problems of previous studies [24–26]. In the current study, the date of the first drug administration was established as the index date for all patients to avoid immortal time bias. A substantial observation period is necessary to assess the development of cancer resulting from medication exposure, because an error can occur in the evaluation of drug-related cancer if the elapsing time after the initial drug exposure is relatively short. Therefore, our study established a latency period of one year before a lung cancer diagnosis for each patient to exclude cancer diagnosis within a short time after the first prescription, to minimize latency time bias as in other studies [14, 15]. To minimize protopathic bias, this study had one-year washout period before the start of an inhaler medication along with a latency period before lung cancer diagnosis.

This study found that the development of lung cancer in COPD was independently associated with a higher Charlson Comorbidity Index and two or more hospitalizations during screening period. Several studies have reported that a high Charlson Comorbidity Index score is an appropriate prognosticator in lung cancer, because of this index’s association with worse survival [27, 28]. However, the explanation for the causal link between Charlson Comorbidity Index and the risk for the development of lung cancer remains unclear.

This study assessed the association between the risk of lung cancer and the severity of COPD by various approaches including a Charlson Comorbidity Index and the number of emergency room visits and hospitalizations. Previous studies reported that emphysema and severe airflow obstruction increased the risk of lung cancer, irrespective of smoking exposure [5, 29, 30]. Frequent hospitalization is also a marker for the severity of COPD [31]. Therefore, our finding that frequent hospitalization was independently associated with the development of lung cancer can be explained by the link between the severity of COPD and frequent hospitalization.

This study found that the development of lung cancer in COPD was independently associated with the presence of DILD. Idiopathic pulmonary fibrosis is an independent risk factor for lung cancer, beyond the effect of smoking [32]. A recent meta-analysis reported the prevalence of lung cancer was 13.74% and incidence rate was 2.07 per 100 person-years in idiopathic pulmonary fibrosis [32]. An even higher prevalence of lung cancer is reported in combined pulmonary fibrosis and emphysema [33]. One study reported that abnormal CT findings of ILD including low attenuation area, fibrosis, and ground glass attenuation and spirometric parameter of FEV_1_/FVC < 70% suggestive of COPD were risk factors for lung cancer, even after adjusting for age, sex, and smoking status [34].

Lung tumorigenesis and fibrosis share common environmental risk factors (i.e., smoking, occupational and environmental exposures) and biological pathways including chronic inflammation, senescence, genetic susceptibility, and epithelial-mesenchymal transition [35, 36]. However, since our finding on the contribution of coexisting DILD to the development of lung cancer in a large COPD cohort has not been previously reported, further investigation is required.

This study has several limitations. First, this was not a prospective study, although the observational design reflects real world clinical practice. Second, because pulmonary function data were not available in the HIRA database, the diagnosis of COPD was based on ICD-10 codes and prescription profiles. Accordingly, the impact of airflow obstruction was not assessed. Third, despite our efforts to exclude asthma as the primary diagnosis, the cohort may still have included patients with asthma, and a lower incidence of lung cancer in asthma may be a potential confounder. Fourth, smoking status, family history of cancer, and the pathologic type of each cancer were not included in the analyses due to lack of information. Fifth, medication adherence was not measured. Sixth, air pollution and socioeconomic factors, such as occupation, were not included in our analysis. Seventh, one of the limitations is the relatively short length of follow-up for identifying a significant effect of inhaler therapy.

## Conclusion

This observational study suggests that coexisting DILD, a higher Charlson Comorbidity Index score, and frequent hospitalization are independently associated with the development of lung cancer, whereas ICS therapy is not protective.

### Supplementary Information

Supplementary Material 1.

## Data Availability

HIRA is an open and public data to which any researcher can get access through the website (https://www.hira.or.kr).

## Abbreviations

ANOVA: One-way analysis of variance
CI: Confidence interval
COPD: Chronic obstructive pulmonary disease
DILD: Diffuse interstitial lung disease
HIRA: Health Insurance Review and Assessment Service
HR: Hazard ratio
ICS: Inhaled corticosteroids
LABA: Long-acting beta-2 agonist
LAMA: Long-acting muscarinic antagonist
OCS: Oral corticosteroid
