# Supplementary material for: The effect of inhaler prescription on the development of lung cancer in COPD: a nationwide population-based study
Source: Respir Res. 2024 May 31;25:229. doi: 10.1186/s12931-024-02838-7 (PMC11140980; doi:10.1186/s12931-024-02838-7)
Supplement: Supplementary file 1 — Supplementary Material 1. [file 12931_2024_2838_MOESM1_ESM.docx]

**Supplementary Table 1.** Result of effect modification analysis

| Variable | Group | HR | 95% CI | | *P*-value | *P*-value  For interaction |
| --- | --- | --- | --- | --- | --- | --- |
| Age group |  |  |  | |  | 0.899 |
| 40 - 49 | LAMA/LABA | Ref. |  | |  |  |
|  | ICS/LABA | 0.93 | 0.26 – 3.32 | | 0.915 |  |
|  | LABA | - |  | | 0.994 |  |
| 50 - 59 | LAMA/LABA | Ref. |  | |  |  |
|  | ICS/LABA | 0.78 | 0.49 – 1.23 | | 0.289 |  |
|  | LABA | 0.59 | 0.08 – 4.29 | | 0.605 |  |
| 60 - 69 | LAMA/LABA | Ref. |  | |  |  |
|  | ICS/LABA | 0.83 | 0.67 – 1.02 | | 0.077 |  |
|  | LABA | 1.54 | 0.93 – 2.55 | | 0.095 |  |
| 70 - 79 | LAMA/LABA | Ref. |  | |  |  |
|  | ICS/LABA | 0.86 | 0.73 – 1.02 | | 0.077 |  |
|  | LABA | 0.89 | 0.52 – 1.51 | | 0.653 |  |
| 80 - | LAMA/LABA | Ref. |  | |  |  |
|  | ICS/LABA | 0.91 | 0.72 – 1.14 | | 0.399 |  |
|  | LABA | 0.94 | 0.44 – 2.01 | | 0.882 |  |
| Sex |  |  |  | |  | 0.504 |
| Male | LAMA/LABA | Ref. |  | |  |  |
|  | ICS/LABA | 0.93 | 0.83 – 1.05 | | 0.255 |  |
|  | LABA | 1.03 | 0.73 – 1.45 | | 0.884 |  |
| Female | LAMA/LABA | Ref. |  | |  |  |
|  | ICS/LABA | 1.03 | 0.76 – 1.40 | | 0.866 |  |
|  | LABA | 1.80 | 0.73 – 4.47 | | 0.205 |  |
| Diffuse Interstitial Lung Disease | | | | | | 0.317 |
| yes | LAMA/LABA | Ref. |  | |  |  |
|  | ICS/LABA | 1.44 | 0.69 – 3.04 | | 0.332 |  |
|  | LABA | - |  | | 0.991 |  |
| no | LAMA/LABA | Ref. |  | |  |  |
|  | ICS/LABA | 0.81 | 0.73 – 0.91 | | **<0.001** |  |
|  | LABA | 1.10 | 0.80 – 1.53 | | 0.556 |  |
| Charlson Comorbidity Index score (CCI) | | | | | | 0.244 |
| CCI = 0 | LAMA/LABA | Ref. |  | |  |  |
|  | ICS/LABA | 1.32 | 0.79 – 2.21 | | 0.285 |  |
|  | LABA | 0.93 | 0.22 – 3.84 | | 0.918 |  |
| CCI = 1 | LAMA/LABA | Ref. |  | |  |  |
|  | ICS/LABA | 0.73 | 0.57 – 0.93 | | **0.010** |  |
|  | LABA | 0.94 | 0.44 – 2.01 | | 0.880 |  |
| CCI = 2 | LAMA/LABA | Ref. |  | |  |  |
|  | ICS/LABA | 0.69 | 0.55 – 0.87 | | **0.002** |  |
|  | LABA | 1.03 | 0.53 – 2.00 | | 0.934 |  |
| CCI ≥ 3 | LAMA/LABA | Ref. |  | |  |  |
|  | ICS/LABA | 0.89 | 0.76 – 1.03 |  | 0.1120 |  |
|  | LABA | 1.22 | 0.78 – 1.90 |  | 0.3941 |  |

Abbreviations: CCI = Charlson Comorbidity Index score; CI = 95% confidence interval; HR = Hazard Ratio; ICS = inhaled corticosteroids; LABA = long-acting beta2-agonist; LAMA = Long-acting muscarinic antagonist; Ref = Reference
